# *Lrit3* Deficient Mouse (*nob6*): A Novel Model of Complete Congenital Stationary Night Blindness (cCSNB)

**DOI:** 10.1371/journal.pone.0090342

**Published:** 2014-03-05

**Authors:** Marion Neuillé, Said El Shamieh, Elise Orhan, Christelle Michiels, Aline Antonio, Marie-Elise Lancelot, Christel Condroyer, Kinga Bujakowska, Olivier Poch, José-Alain Sahel, Isabelle Audo, Christina Zeitz

**Affiliations:** 1 INSERM, U968, Paris, France; 2 CNRS, UMR_7210, Paris, France; 3 Sorbonne Universités, UPMC Univ Paris 06, UMR_S 968, Institut de la Vision, Paris, France; 4 Centre Hospitalier National d'Ophtalmologie des Quinze-Vingts, INSERM-DHOS CIC 503, Paris, France; 5 Massachusetts Eye and Ear Infirmary, Ocular Genomics Institute, Boston, Massachusetts, United States of America; 6 Laboratoire de Bioinformatique Intégrative et Génomique, ICube, CNRS, UMR_7357, Strasbourg, France; 7 Institute of Ophthalmology, University College of London, London, United Kingdom; 8 Fondation Ophtalmologique Adolphe de Rothschild, Paris, France; 9 Académie des Sciences–Institut de France, Paris, France; University Zürich, Switzerland

## Abstract

Mutations in *LRIT3*, coding for a Leucine-Rich Repeat, immunoglobulin-like and transmembrane domains 3 protein lead to autosomal recessive complete congenital stationary night blindness (cCSNB). The role of the corresponding protein in the ON-bipolar cell signaling cascade remains to be elucidated. Here we genetically and functionally characterize a commercially available *Lrit3* knock-out mouse, a model to study the function and the pathogenic mechanism of LRIT3. We confirm that the insertion of a *Bgeo/Puro* cassette in the knock-out allele introduces a premature stop codon, which presumably codes for a non-functional protein. The mouse line does not harbor other mutations present in common laboratory mouse strains or in other known cCSNB genes. *Lrit3* mutant mice exhibit a so-called *no b-wave* (*nob*) phenotype with lacking or severely reduced b-wave amplitudes in the scotopic and photopic electroretinogram (ERG), respectively. Optomotor tests reveal strongly decreased optomotor responses in scotopic conditions. No obvious fundus auto-fluorescence or histological retinal structure abnormalities are observed. However, spectral domain optical coherence tomography (SD-OCT) reveals thinned inner nuclear layer and part of the retina containing inner plexiform layer, ganglion cell layer and nerve fiber layer in these mice. To our knowledge, this is the first time that SD-OCT technology is used to characterize an animal model for CSNB. This phenotype is noted at 6 weeks and at 6 months. The stationary *nob* phenotype of mice lacking *Lrit3*, which we named *nob6*, confirms the findings previously reported in patients carrying *LRIT3* mutations and is similar to other cCSNB mouse models. This novel mouse model will be useful for investigating the pathogenic mechanism(s) associated with *LRIT3* mutations and clarifying the role of LRIT3 in the ON-bipolar cell signaling cascade.

## Introduction

Congenital stationary night blindness (CSNB) is a clinically and genetically heterogeneous group of non-progressive retinal disorders caused by mutations in genes implicated in the phototransduction cascade or in retinal signaling from photoreceptors to adjacent bipolar cells [1]. These disorders can be associated with other ocular abnormalities, including reduced visual acuity, myopia, nystagmus and strabismus. Most of the individuals affected with CSNB show a characteristic electroretinogram (ERG) response, named Schubert-Bornschein, in which the b-wave amplitude is smaller than that of the a-wave in the dark-adapted bright flash condition [2]. This electronegative waveform can be divided in two subtypes, incomplete (ic)CSNB and complete (c)CSNB [3]. cCSNB is characterized by a drastically reduced rod b-wave response due to ON-bipolar cell dysfunction, and specific cone ERG waveforms [4]. cCSNB has been associated with mutations in *NYX* [5], [6], *GRM6* [7], [8], *TRPM1* [9]–[11] and *GPR179* [12], [13], genes expressed in the inner nuclear layer (INL) of the retina [6], [14]–[17] and coding for proteins localized at the dendritic tips of ON-bipolar cells [12], [14], [15], [17]–[20]. Recently, we have identified mutations in *LRIT3*, a gene coding for a Leucine-Rich Repeat (LRR), immunoglobulin-like and transmembrane domains 3 protein, that lead to cCSNB [21]. The corresponding protein also localizes at the dendritic tips of ON-bipolar cells in the retina [21].

Animal models have been shown to be an excellent tool for identifying and elucidating the pathogenic mechanism(s) of gene defects underlying CSNB [15], [18], [22]–[32]. Clinically, the phenotypes of these models can be assessed as in patients by performing full-field electroretinography, fundus autofluorescence imaging (FAF), and optical coherence tomography (OCT). In addition, the retinal structure can be investigated *post mortem* in affected animals and compared to unaffected littermates. This aspect is valuable for a better assessment of histological changes since access to human retinas remains extremely difficult. Various mouse models have been design or are naturally occurring for Schubert-Bornschein type of CSNB with dysfunction in molecules important for the signaling from the photoreceptors to the adjacent bipolar cells. Six mouse models with four different gene defects, *Nyx* (no b-wave (*nob*)), *Grm6* (*nob3* and *nob4*), *Trpm1* and *Gpr179* (*nob5*) have already been published for cCSNB [12], [15], [18], [25], [26], [32]–[36].

These mouse models were helpful in dissecting the ON-bipolar cell signaling cascade. In darkness, the glutamate neurotransmitter released by the photoreceptors binds to the metabotropic glutamate receptor 6 (GRM6/mGluR6) [16], [37]. This binding leads to the activation of the α-subunit of the G-protein, G_0_α [38], [39], which results in the closure of the non-selective cation channel TRPM1. When the photoreceptors are stimulated by light, the deactivation of the G-protein in the ON-bipolar cells is responsible for the opening of TRPM1, resulting in the formation of the ERG b-wave [15], [25], [32]. GRM6 and NYX interact with TRPM1 and are essential for its localization at the dendritic tips of ON-bipolar cells [23], [24]. In addition GPR179 is essential for the action of the G-protein downstream of mGluR6 via the correct localization of Regulator of G protein Signaling proteins (RGS) [20], GPR179 also interacts with both mGluR6 and TRPM1 and its correct localization is mediated through mGluR6 [40].

The exact role of LRIT3 in this cascade remains to be elucidated. As previously described, NYX is essential for the correct localization of TRPM1 [23]. However, NYX alone would not be sufficient to bring TRPM1 at the dendritic tips of ON-bipolar cells in the retina. Mouse and human NYX are mainly extracellular proteins [41], [42] that lack an intracellular PDZ-binding domain important for binding to scaffolding proteins involved in membrane trafficking [43]. Thus, another transmembrane protein is needed to interact with the scaffolding proteins for TRPM1 localization [23]. As LRIT3 resembles proteins of the SALM family, in particular containing a PDZ-binding motif, our hypothesis is that LRIT3 might interact with scaffolding proteins to bring TRPM1 to the cell surface and thereafter LRIT3 together with NYX might hold the channel in this form [21]. We have not so far been able to confirm this hypothesis or to study the pathogenic mechanism(s) of *LRIT3* defects underlying cCSNB by *in vitro* experiments due to the lack of an antibody able to detect human LRIT3 at the cell surface of transfected cells and the lack of a characterized mouse model for *Lrit3*, which will be named *nob6*.

The aim of this study was to characterize a commercially available *Lrit3* mouse model and to establish whether this animal would be a reliable model for human cCSNB.

## Materials and Methods

### Ethics statements

All animal procedures were performed according to the Association for Research in Vision and Ophthalmology (ARVO) Statement for the Use of Animals in Ophthalmic and Visual Research and were approved by the French Minister of Agriculture (authorization A-75-1863 delivered on 09^th^ November 2011). All efforts were made to minimize suffering.

### *Lrit3* cDNA sequence

We deposited at GenBank the experimentally validated cDNA sequence of *Lrit3* (BankIt1682729 *Lrit3* KF954709), which corresponds to the mouse *Lrit3* cDNA sequence, which was updated on 10^th^ December 2013 (NM_001287224.1).

### Animal Care

Three 129/SvEv-C57BL/6 heterozygous knock-out mice for *Lrit3* of each sex were obtained from a company (TF2034, Taconic, Hudson, NY). These mice were intercrossed (Centre d'Exploration et de Recherche Fonctionnelle Expérimentale CERFE, Evry, France) to produce wild-type (*Lrit3* ^+/+^), heterozygous (*Lrit3^nob6^* ^/+^) and mutant (*Lrit3^nob6/nob6^*) offspring. To follow up the phenotype at different ages, the two time points, six weeks and six months, were selected. For the optomotor test, we used seven *Lrit3^+/+^*, eleven *Lrit3^nob6/+^*, nine *Lrit3^nob6/nob6^* mice of six weeks and nine *Lrit3^+/+^*, eight *Lrit3^nob6/+^* and nine *Lrit3^nob6/nob6^* mice of six months. The same animals were used for ERG recordings, FAF, and Spectral-Domain Optical Coherence Tomography (SD-OCT) except for one six weeks *Lrit3^+/+^* and one six weeks *Lrit3^nob6/nob6^* who died during FAF. For histology, two animals of each genotype for both ages were used. Mice were housed in a temperature-controlled room with a 12-h light/12-h dark cycle. Fresh water and rodent diet were available *ad libitum*.

### Polymerase chain reaction (PCR) genotyping for *Lrit3*

DNA was isolated from mouse tails with 50 mM NaOH after incubation at 95°C for 30 min. Two couples of primers were designed to amplify wild-type (wt) or mutant allele independently (HOT FIREPol, Solis Biodyne, Tartu, Estonia): mLrit3_4aF and mLrit3_4aR for the wt allele, mLrit3_3F and mLrit3_CasR for the mutant one (Table S1). PCR products were separated by electrophoresis on 2% agarose gels, stained with ethidium bromide, and visualized using the Gel Doc XR+ system (Bio-Rad, Hercules, CA).

### Genotyping for common mutations found in laboratory mouse strains

The genotyping for the *Crb1^rd8^* mutation was carried out by qPCR Taqman on genomic DNA with probes specific for wt or mutant allele, respectively (Table S2). The presence of common mutations in laboratory strains *Pde6β^rd1^*, *Gnat2^cpfl3^*, and c.230G>T p.Arg77Leu in *Tyr* were investigated by direct Sanger sequencing. The following primers were used: Pde6b_7–8F and Pde6b_7–8R for the substitution in *rd1* (Gotaq DNA Polymerase, Promega, Madison, WI, USA), Pde6b_G2shortF and Pde6b_G1shortR for the insertion in *rd1* (HOT FIREPol), Gnat2_6F and Gnat2_7R for the muration in *cpfl3* (HOT FIREPol) and Tyr_F and Tyr_R for p.Arg77Leu in *Tyr* (HOT FIREPol) (Tables S3, S4, S5). Subsequently, PCR products were Sanger sequenced with a sequencing mix (BigDyeTerm v1.1 CycleSeq kit, Applied Biosystems, Courtabœuf, France), analyzed on an automated 48-capillary sequencer (ABI 3730 Genetic analyzer, Applied Biosystems), and the results interpreted by applying a software (SeqScape, Applied Biosystems).

### Genotyping for genes with mutations underlying cCSNB

DNA of six founder mice were used to sequence the flanking intronic and exonic sequences of *Grm6*, *Gpr179*, *Nyx*, *Lrit3* and *Trpm1* as well as intron 2 of *Grm6* and intron 1 of *Gpr179* (HOT FIREPol). The corresponding primers, fragment sizes and annealing temperatures used are reported in Tables S1 and S6, S7, S8, S9. Identified variants were evaluated in respect to the conservation (UCSC Genome Browser: http://genome.ucsc.edu/), pathogenicity predictions (Sorting Intolerant from Tolerant (SIFT): http://sift.bii.a-star.edu.sg/, and PolyPhen-2: http://genetics.bwh.harvard.edu/pph2/) and presence in mouse strains used to generate the *Lrit3* mouse model (Ensembl Genome Browser: http://www.ensembl.org/index.html).

### Electroretinography

Electroretinography was performed according to Yang and co-workers with some modifications [44]. After overnight dark adaptation, mice were anesthetized with ketamine (80 mg/kg) and xylazine (8 mg/kg). Eye drops were used to dilate the pupils (0.5% mydriaticum, 5% neosynephrine) and anesthetize the cornea (0.4% oxybuprocaine chlorhydrate). Body temperature was maintained at 37°C through the use of a circulating hot water heating pad. Upper and lower lids were retracted to keep the eyes open and bulging. Contact lens electrodes for mice (Mayo Corporation, Japan) were placed on the corneal surface to record ERG. Needle electrodes placed subcutaneously in cheeks served as reference and a needle electrode placed in the back served as ground. Recordings were made from both eyes simultaneously. The light stimulus was provided by a 150 Watt xenon lamp in a Ganzfeld stimulator (Multilinear Vision, Jaeger Toennies, Germany). Responses were amplified and filtered (1 Hz-low and 300 Hz-high cut off filters) with a 1 channel DC-/AC-amplifier. Eight levels of stimulus intensity ranging from 0.0006 cd.s/m^2^ to 60 cd.s/m^2^ were used for the dark-adapted ERG recording. Each scotopic ERG response represents the average of five responses from a set of five flashes of stimulation. To isolate cone responses a 10-minute light adaptation at 20 cd/m^2^ was used to saturate rod photoreceptors. Six levels of stimulus intensities ranging from 0.3 cd.s/m^2^ to 60 cd.s/m^2^ were used for the light-adapted ERGs. The light-adapted ERGs were recorded on the same rod-suppressive white background as for the light adaptation. Each cone photopic ERG response represents the average of twenty responses to a set of twenty consecutive flashes. The major components of the ERG were measured conventionally. The a-wave amplitude was measured from the baseline to the a-wave trough and the b-wave amplitude was measured from the a-wave trough to the peak of the b-wave or, if no a-wave was present, from the baseline. Implicit times were measured from the onset of the flash stimulus to the a-wave trough and the b-wave peak, respectively.

### Optomotor response

Optomotor test was performed as previously described [45]. After overnight dark adaptation, mice were placed on a grid platform (11.5 cm diameter, 19 cm above the bottom of the drum) surrounded by a motorized drum (29 cm diameter) that could be revolved clockwise or anticlockwise at two revolutions per minute. Vertical black and white stripes of a defined spacial frequency were presented to the animal. Spatial frequencies tested were 0.063, 0.125, 0.25, 0.5 and 0.75 cycles per degree. The stripes were rotated for 1 min in each direction with an interval of 10 sec between the two rotations. Animals were videotaped using a digital video camera for subsequent scoring of head movements. Tests were initially performed under scotopic conditions, using the night shot function of the camera. Mice were then subjected to two lamps of 60 Watt each for 5 min and photopic measurements were performed. Head movements were scored only if the angular speed of the movement corresponded to that of the drum rotation. Head movements in both directions were averaged to obtain the number of head movements per minute.

### FAF

Photographs of the eye fundus and autofluorescence were obtained with a scanning laser ophthalmoscope (SLO) (HRA1, Heidelberg, Germany). Mouse pupils were dilated by the ocular instillation of 0.5% mydriaticum and 5% neosynephrine.

### SD-OCT

Mice were anesthetized by isoflurane inhalation and maintained under anesthesia via a mask. Eye drops were used to dilate the pupils (0.5% mydriaticum, 5% neosynephrine) and eye dehydration was prevented by regular instillation of sodium chloride drops. SD-OCT images were recorded for both eyes using a spectral domain ophthalmic imaging system (Bioptigen, Inc., Durham, NC, USA). We performed rectangular scans consisting of a 1.4 mm by 1.4 mm perimeter with 1000 A-scans per B-scan with a total B-scan amount of 100. Scans were obtained first while centered on the optic nerve, and then with the nerve displaced either temporally/nasally or superiorly/inferiorly. SD-OCT scans were exported from InVivoVue as AVI files. These files were loaded into ImageJ (version 1.47; National Institutes of Health, Bethesda, MD) where they were registered using the Stackreg plug-in. If the optic nerve was placed temporally/nasally, three B-scans at the level of the nerve were averaged and measurements were performed 500 µm away from the optic disc, on each side. In the case where the optic nerve was placed superiorly/inferiorly, 3 B-scans placed 500 µm away from the optic disc were averaged to perform the measurements. We measured the thickness of outer nuclear layer (ONL), INL and a complex comprising inner plexiform layer (IPL), ganglion cell layer (GCL) and nerve fiber layer (NFL) that we called IPL+GCL+NFL [46].

### Preparation of retinas for RNA

Mice were killed by CO_2_ administration and cervical dislocation. Eyes were removed and dissected in PBS to collect one retina of each mouse. Retinas were soaked in RNA latter (Qiagen, Venlo, The Netherlands) and stored at −80°C until used.

### RT-PCR

Total RNA was isolated from retinas of 6 weeks old mice using a kit (RNeasy Mini Kit, Qiagen) and 500 ng were used to synthesize cDNA with a reverse transcriptase (SuperScript II, Invitrogen, Carlsbad, CA, USA), according to the manufacturer's protocol. Two couples of primers were designed to amplify wt or mutant cDNA independently: mLrit3_RT_ex2F 5′ GTGGAGCTGCAGTACCTCT 3′ and mLrit3_RT_ex3R 5′ GCTGACATCATCACGGACG 3′ for the wt cDNA, mLrit3_RT_ex2F and mLrit3_RT_CasR 5′ GCTCGAAGCTTATCGCTAGT 3′ for the mutant one. Amplification was carried out by a DNA polymerase (HOT FIREPol).

### Preparation of retinal sections for histology

Mice were killed by CO_2_ administration and cervical dislocation. Eyes were removed, fixed in Davidson's fixative (22% formalin 37%, 33% absolute ethanol, 11% glacial acetic acid) at room temperature for 3 h, dehydrated and embedded in paraffin. Sections of 5-µm-thickness were cut on a microtome (HM 340E, Microm Microtech, Francheville, France), mounted onto Superfrost plus glass slides (Thermo Fisher Scientific, Waltham, MA, USA), dried in an oven and stored at room temperature until used.

### Histology

Retinal sections for histology were hematoxylin-eosin colored by an automaton (HMS 70, Microm Microtech), dried and mounted with a non-aqueous medium (Diamount, Diapath, Martinengo, BG, Italy). Slides were then scanned with a Nanozoomer 2.0 high throughput (HT) equipped with a 3-charge–coupled device time delay integration (TDI) camera (Hamamatsu Photonics, Hamamatsu, Japan).

### Statistical analyses

Statistical analyses were performed using the statistical software (SPSS, version 19.0 Inc, Chicago, Illinois, USA). Kruskal-Wallis's test was used to compare head movements per minute in optomotor test and retinal layer thickness in SD-OCT among the three genotypes. *Post-hoc* comparisons were used to compare the genotypes two by two when the Kruskal-Wallis's test permitted to reject the hypothesis H0. These *post-hoc* analyses were also applied to compare results obtained from mice at six weeks and six months of ages. The number of animals used for the different phenotyping experiments and groups are described above (Animal Care). Tests were considered as significant when p<0.05.

## Results

### Genetic characterization of the *Lrit3* deficient mouse

**Figure 1:**
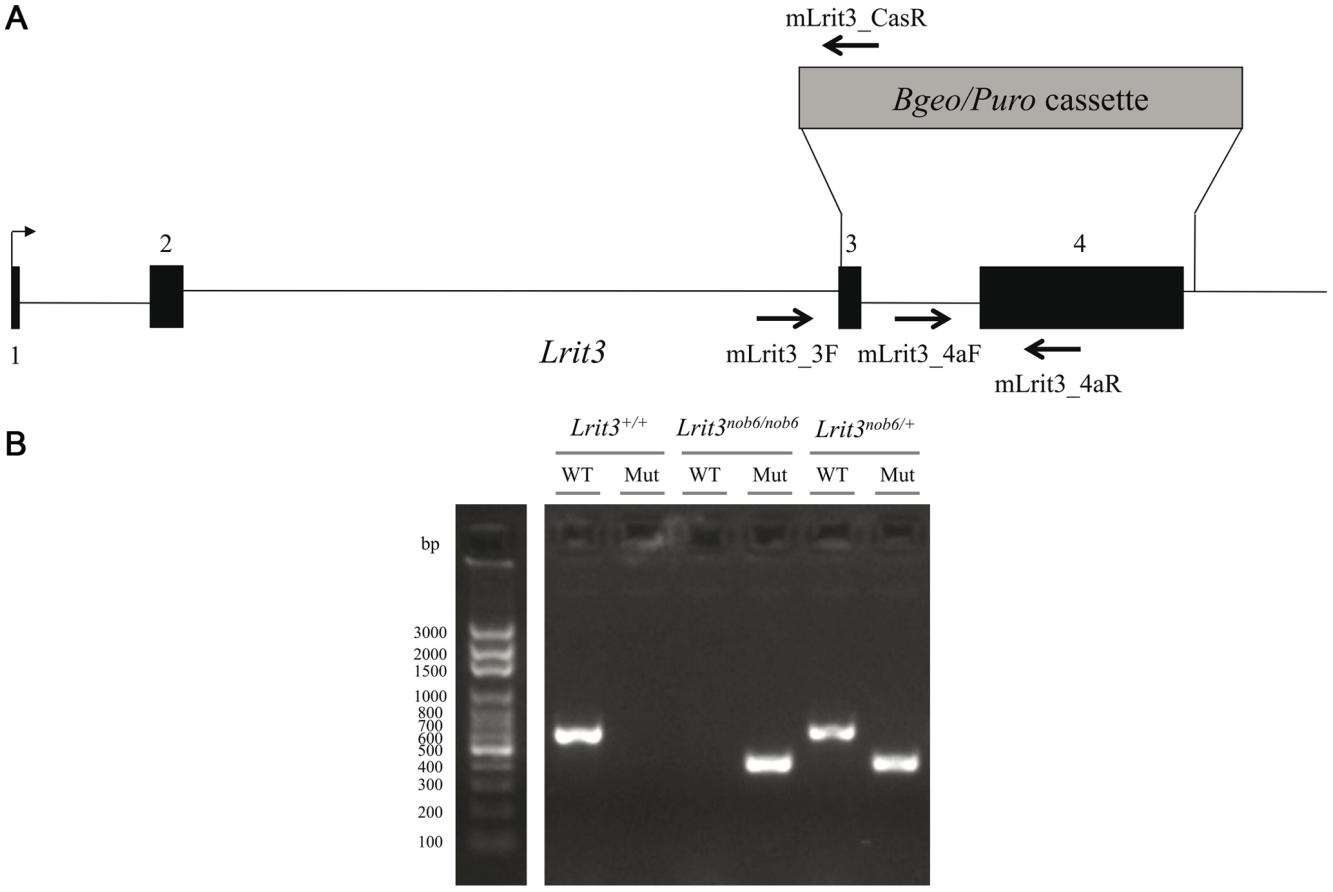
Construction of the *Lrit3* knock-out allele and genotyping. (A) Wild-type (wt) *Lrit3* allele comprises 4 exons. In the knock-out (ko) construction, exons 3 and 4 were replaced by a selection cassette with only 21 bp of exon 3 still remaining. For genotyping, mLrit3_ex4aF and mLrit3_ex4aR were designed to only amplify the wt allele, whereas mLrit3_ex3F and mLrit3_CasR were designed to only amplify the ko allele. (B) After migration on 2% agarose gel, *Lrit3^+/+^* mice exhibited a single fragment at the expected length of 602 bp, *Lrit3^nob6/nob6^* exhibited a single fragment at the expected length of 377 bp and *Lrit3^nob6/+^* mice exhibited both fragments. Legends: WT: wild-type allele; Mut: mutant allele.

To obtain an *in vivo* tool to study the pathogenic mechanism(s) of cCSNB due to mutations in *LRIT3*, common databases were checked for the existence of commercially available mice lacking functional LRIT3. Indeed, *Lrit3* knock-out mouse line (LEXKO-2034) was generated by a company (Lexicon Pharmaceuticals, The Woodlands, TX, USA) and a basic phenotype description was given. For this line no obvious phenotype had been noted (behavior, hematology, endocrinology, immunology, cardiology, radiology, fertility, ophthalmology). Since the ophthalmic examination was also inconspicuous and no information on the phenotyping protocol was available, we wanted to elucidate if these mice represent a model for cCSNB. Six 129/SvEv-C57BL/6 mice heterozygous knock-out for *Lrit3* of both sexes were obtained from a company (Taconic, Hudson, NY, USA) and three breeding pairs were established. For the knock-out allele, exons 3 and 4 are deleted and replaced by a *Bgeo/Puro* cassette, with only 21 bp of exon 3 remaining (Figure 1A). These mice were intercrossed and the offspring was genotyped by size specific PCR strategies. While *Lrit3^+/+^* and *Lrit3^nob6/nob6^* exhibited a single fragment at 602 bp and 377 bp respectively, *Lrit3^nob6/+^* mice revealed both fragments (Figure 1B). *Lrit3* mice used in these experiments were free of common mutations in *Tyr* [47], *Crb1* [48], *Pde6β* [49], [50] and *Gnat2* [51] frequently found in laboratory strains and associated with different eye phenotypes (data not shown). Since already three naturally occurring mouse models for cCSNB have been described (*Nyx* [34], *Gpr179* [12] and *Grm6* [35]), we excluded these mutations and mutations in all genes underlying cCSNB (*Nyx*, *Grm6*, *Trpm1*, *Gpr179* and *Lrit3*) by a direct sequencing approach. Only 129/SvEv and C57BL/6 strain specific non-pathogenic variants were detected (Tables S10, S11).

### Validation of the *Lrit3* deficient mouse

**Figure 2:**
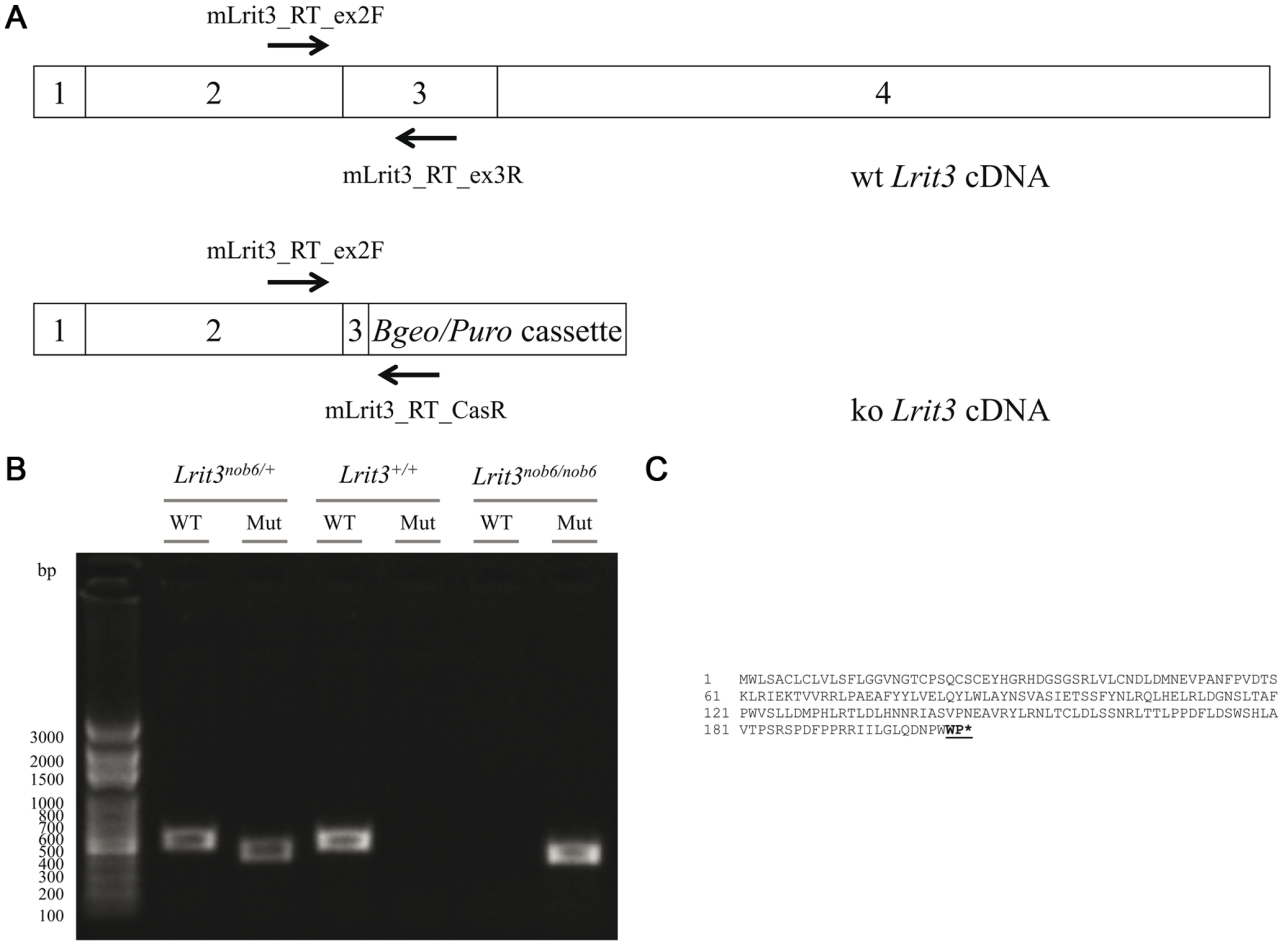
wt and ko *Lrit3* cDNAs and RT-PCR. (A) ko cDNA comprised the remaining 21 bp of exon 3 and only the first 8 bp of the selection cassette which leads to a premature stop codon. mLrit3_RT_ex2F and mLrit3_RT_ex3R were designed to only amplify the wt cDNA whereas mLrit3_RT_ex2F and mLrit3_RT_CasR were designed to only amplify the supposed ko cDNA. (B) After amplification and migration on agarose gel, *Lrit3^+/+^* mice exhibited a single fragment at 539 bp, *Lrit3^nob6/nob6^* mice exhibited a 443 bp fragment and *Lrit3^nob6/+^* mice exhibited both fragments. Legends: WT: wild-type cDNA; Mut: mutant cDNA. (C) Sequence of the ko LRIT3 protein. This 206 amino acid protein are supposed to lack its Ig-like, Serine-rich, fibronectin III, transmembrane and PDZ-binding domains.

*Lrit3* deficient mice were validated at transcript level by performing RT-PCR experiments with subsequent direct sequencing of the amplicons (Figure 2A–B). A 539 bp and a 443 bp fragment was obtained for the wt and the mutant cDNA, respectively. Heterozygous mice exhibited both fragments confirming the PCR genotyping for *Lrit3* model (Figure 2B). The knock-out allele for *Lrit3* produced a transcript including 21 bp of exon 3 and the first 8 bp of the selection cassette (c.611_2046delinsGGCCATAG), which leads to a premature stop codon (p.Phe204Trpfs*3) (Figure 2A). So, if a protein is produced, it would code for a short 206 amino acid lacking presumably the Immunoglobulin-like (Ig-like), Serine-rich, fibronectin III, transmembrane and PDZ-binding domains (Figure 2C).

### Functional characterization of mice lacking functional LRIT3

#### Electroretinography

**Figure 3:**
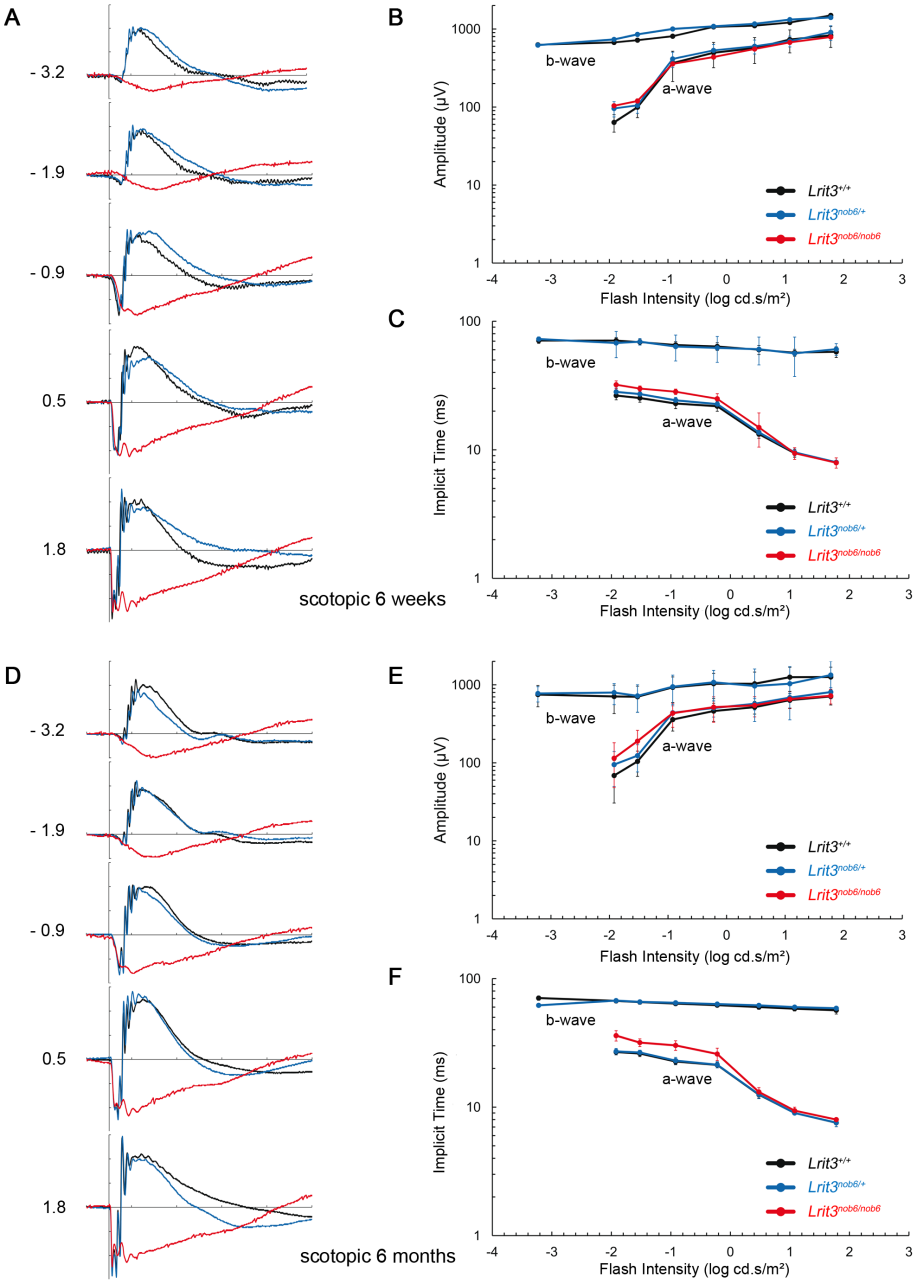
Scotopic ERG phenotype. Dark-adapted ERG series were obtained from representative *Lrit3^+/+^* (black line), *Lrit3^nob6/+^* (blue line) and *Lrit3^nob6/nob6^* (red line) littermates. (A) At 6 weeks of age. The scale indicates 100 ms and 200 µV. Values to the left of the row of waveforms indicate flash intensity in log cd.s/m^2^. (B) Amplitude of the major components of the dark-adapted ERG with increasing flash intensity at 6 weeks of age. The b-wave component is absent in *Lrit3^nob6/nob6^* mice and therefore this data is not plotted. (C) Implicit time of the major components of the dark-adapted ERG with increasing flash intensity at 6 weeks of age. The b-wave component is absent in *Lrit3^nob6/nob6^* mice and therefore this data is not plotted. (D) At 6 months of age. The scale indicates 100 ms and 200 µV. Values to the left of the row of waveforms indicate flash intensity in log cd.s/m^2^. (E) Amplitude of the major components of the dark-adapted ERG with increasing flash intensity at 6 months of age. The b-wave component is absent in *Lrit3^nob6/nob6^* mice and therefore this data is not plotted. (F) Implicit time of the major components of the dark-adapted ERG with increasing flash intensity at 6 months of age. The b-wave component is absent in *Lrit3^nob6/nob6^* mice and therefore this data is not plotted.

**Figure 4:**
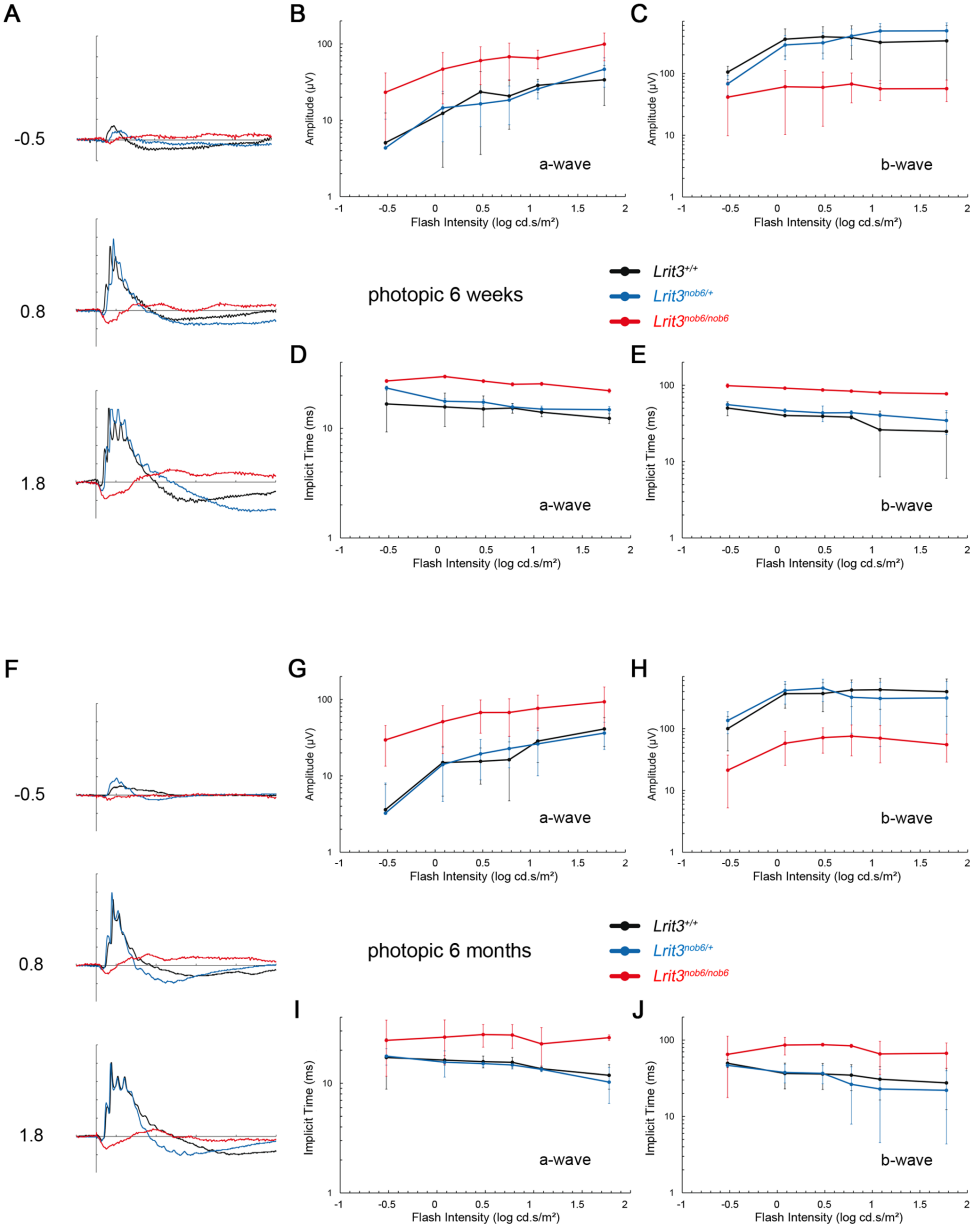
Photopic ERG phenotype. Cone-mediated ERG series were obtained from representative *Lrit3^+/+^* (black line), *Lrit3^nob6/+^* (blue line) and *Lrit3^nob6/nob6^* (red line) littermates. (A) At 6 weeks of age. The scale indicates 100 ms and 100 µV. Values to the left of the row of waveforms indicate flash intensity in log cd.s/m^2^. (B) Amplitude of the a-wave with increasing flash intensity at 6 weeks of age. (C) Amplitude of the b-wave with increasing flash intensity at 6 weeks of age. (D) Implicit time of the a-wave with increasing flash intensity at 6 weeks of age. (E) Implicit time of the b-wave with increasing flash intensity at 6 weeks of age. (F) At 6 months of age. The scale indicates 100 ms and 100 µV. Values to the left of the row of waveforms indicate flash intensity in log cd.s/m^2^. (G) Amplitude of the a-wave with increasing flash intensity at 6 months of age. (H) Amplitude of the b-wave with increasing flash intensity at 6 months of age. (I) Implicit time of the a-wave with increasing flash intensity at 6 months of age. (J) Implicit time of the b-wave with increasing flash intensity at 6 months of age.

ERG responses of *Lrit3^+/+^*, *Lrit3^nob6/+^* and *Lrit3^nob6/nob6^* mice were recorded at six weeks and at six months under scotopic and photopic conditions and increasing flash intensities. At six weeks of age, under scotopic conditions, which are dominated by rod-pathway function, *Lrit3^+/+^* mice showed normal responses with the classic positive deflection of the b-wave. As expected, with increasing flash intensities, amplitudes of both a-wave and b-wave increased whereas implicit times of both waves shortened (Figure 3A–C). ERG responses of heterozygous mice were undistinguishable from the *Lrit3^+/+^* responses (Figure 3A–C). In contrast, *Lrit3^nob6/nob6^* mice were lacking b-wave on their ERG responses, while a-waves were comparable in amplitude or implicit time to *Lrit3^nob6/+^* and to *Lrit3^+/+^* mice (Figure 3A–C). This led to an electronegative ERG waveform in *Lrit3^nob6/nob6^* mice, in which the b-wave was absent while the a-wave was preserved, indicating a signal transmission defect between rod photoreceptors and ON-bipolar cells, whereas the phototransduction in rod photoreceptors is not affected. At 6 weeks of age, under photopic conditions, which reflect cone circuitry function, *Lrit3^+/+^* mice showed normal ERG responses with the classic positive deflection of the b-wave. As expected, with increasing flash intensities, the amplitudes of both, the a-wave, when measurable, and the b-wave increased, whereas implicit times of both waves shortened (Figure 4A–E). Responses in *Lrit3^nob6/+^* mice were not different from those of *Lrit3^+/+^* (Figure 4A–E). In contrast, ERG responses for *Lrit3^nob6/nob6^* were very variable and showed larger a-wave amplitudes, shorter b-wave amplitudes and longer implicit times than for *Lrit3^+/+^* mice (Figure 4A–E). These results are in keeping with cone-mediated pathway dysfunction in these mice. The *nob* phenotype observed in mutant mice seemed to be stationary since no difference was observed between responses obtained at six weeks (Figures 3A–C and 4A–E) and six months (Figures 3D–F and 4F–J). This new *nob* mouse model was named *nob6*.

#### Optomotor test

**Figure 5:**
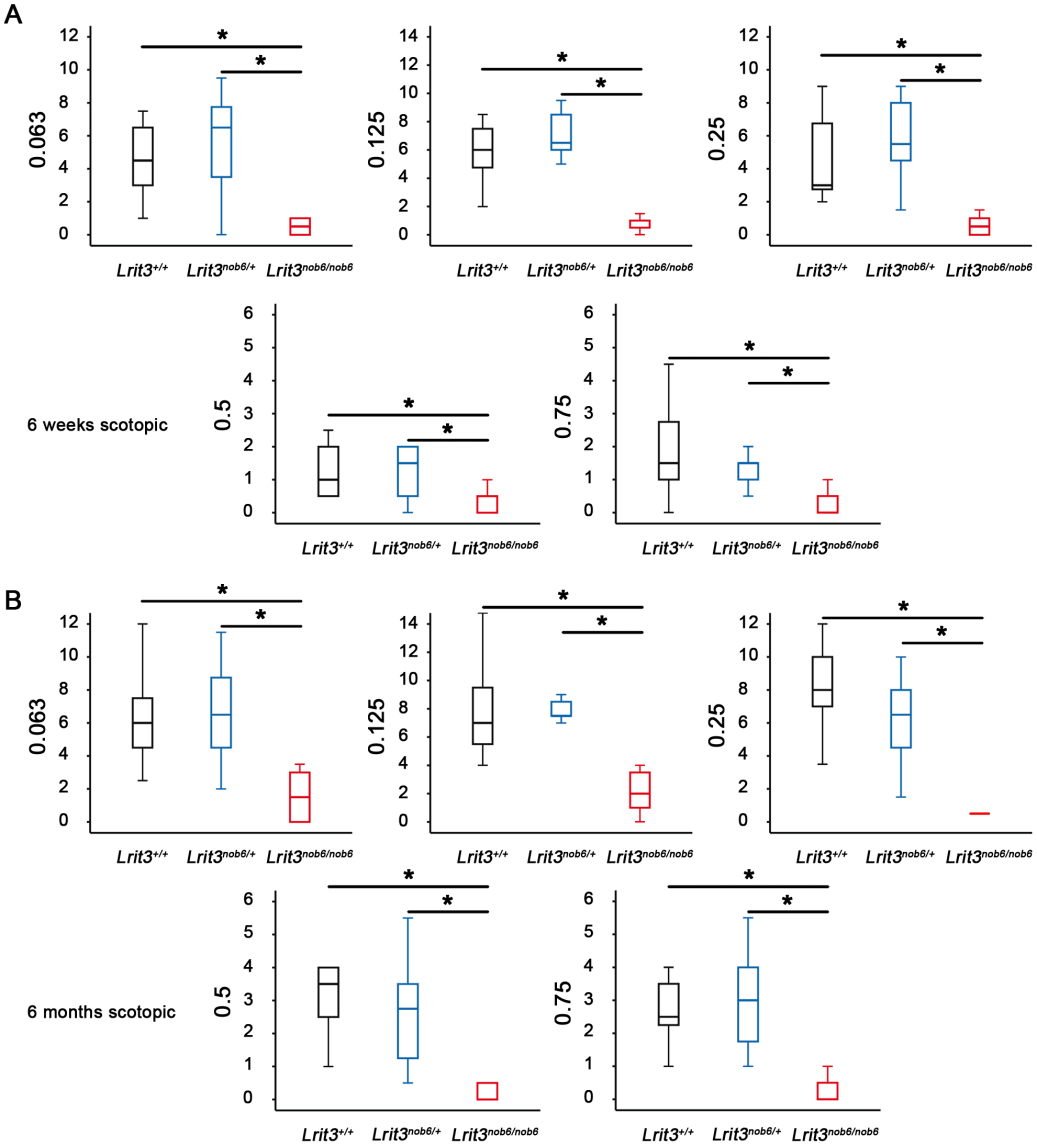
Optomotor responses. The number of head movements per minute was obtained in scotopic conditions with spatial frequencies from 0.063 to 0.75-Wallis statistical test in representative *Lrit3^+/+^* (black box), *Lrit3^nob6/+^* (blue box) and *Lrit3^nob6/nob6^* (red box) littermates. The star indicates a significant test (p<0.05). (A) At 6 weeks of age. (B) At 6 months of age.

We tested optomotor responses of *Lrit3^+/+^*, *Lrit3^nob6/+^* and *Lrit3^nob6/nob6^* mice at 6 weeks and 6 months of age under scotopic and photopic conditions at increasing spatial frequency. Under scotopic conditions, for all the genotypes at both ages, a maximum number of head movements per minute was reached at 0.125 cpd. The number of head movements per minute decreased then with increasing spatial frequency but the exact visual acuity was not evaluable because the zero was never reached. Responses in heterozygous mice were globally undistinguishable from the *Lrit3^+/+^* responses (Figure 5A–B). However, optomotor responses in *Lrit3^nob6/nob6^* mice were statistically decreased at all spatial frequencies and at both ages (from p<0.001 to p = 0.032) (Figure 5A–B). Moreover, no statistical differences were observed for the mutant mice responses between 6 weeks and 6 months. Analyses of the optomotor responses under photopic conditions were not conclusive and therefore the data are not shown.

#### FAF

To validate if *Lrit3^nob6/nob6^* mice represent indeed a good model for a stationary not progressive night blindness disease, which is not associated with striking fundus abnormalities, we performed FAF. In the retinal pigment epithelium (RPE) of mammals, lipofuscin (LF) accumulates as a result of outer segment renewal. LF levels increase with age and can be modified in case of photoreceptor/RPE diseases [52], [53]. This dynamic process can be imaged through FAF. None of the six weeks or six months old mice whatever their genotypes exhibited changes in FAF (data not shown) in keeping with the absence of photoreceptor/RPE disease.

#### SD-OCT and histology

**Figure 6:**
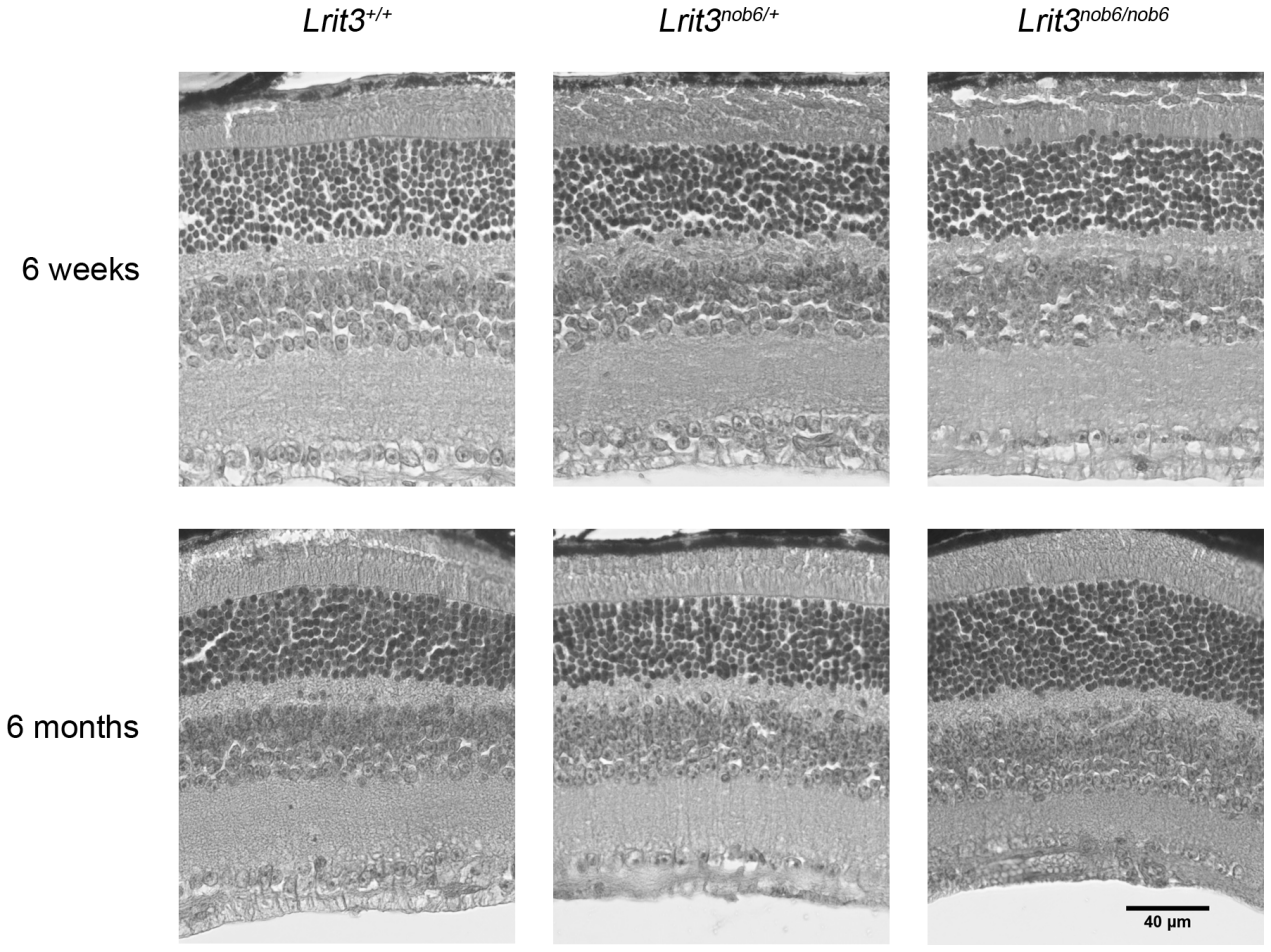
Retinal anatomy of *Lrit3^nob6/nob6^* mice. Retinal sections of representative *Lrit3^+/+^*, *Lrit3^nob6/+^* and *Lrit3^nob6/nob6^* littermates were compared by light microscopy at 6 weeks and 6 months of age. Scale bar, 40 µm.

**Figure 7:**
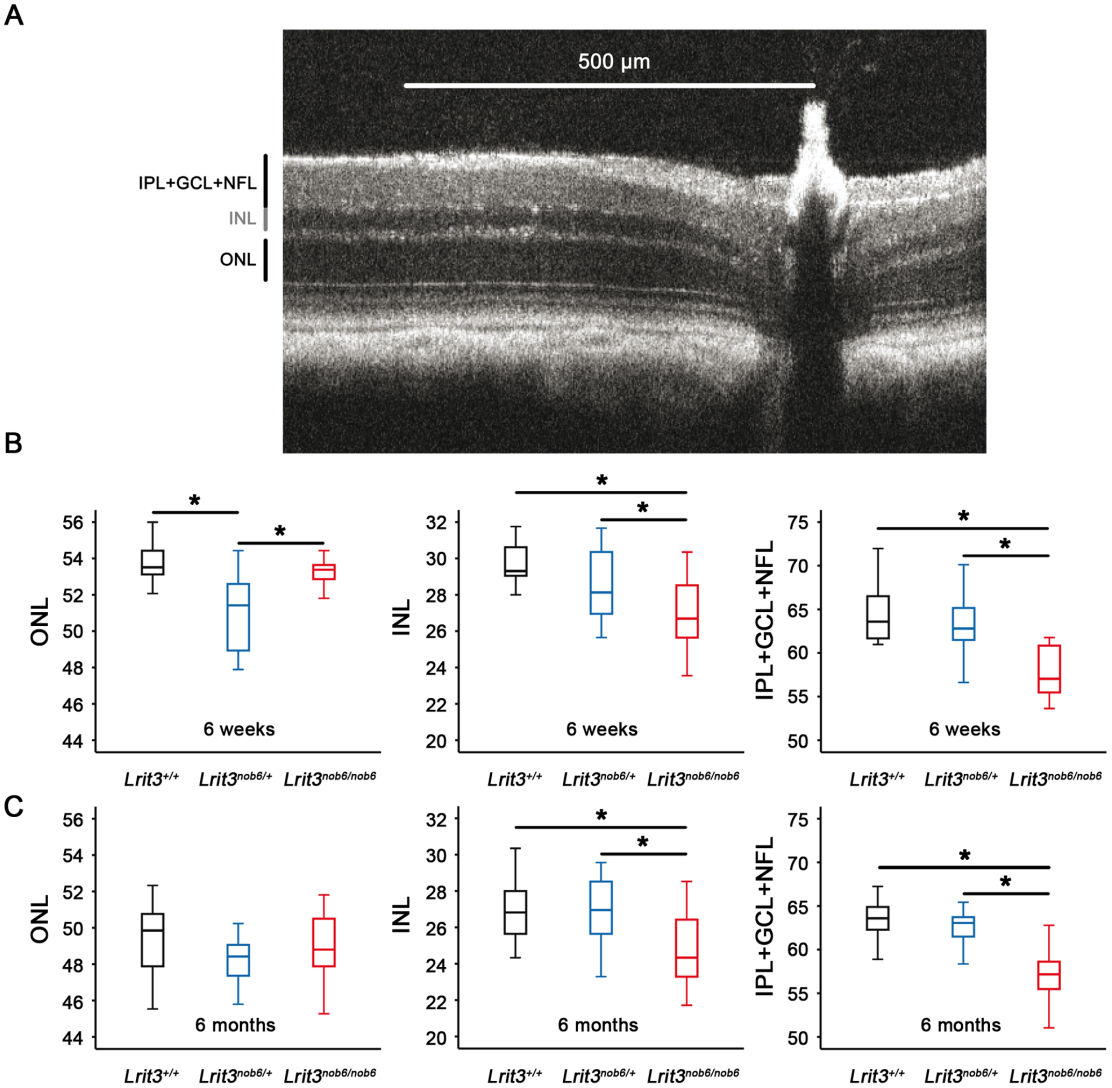
SD-OCT retinal nuclear layers measurements. (A) ONL, INL and IPL+GCL+NFL thickness were obtained by SD-OCT and compared using Kluskal-Wallis statistical test in representative *Lrit3^+/+^* (black box), *Lrit3^nob6/+^* (blue box) and *Lrit3^nob6/nob6^* (red box) littermates. The star indicates a significant test (p<0.05). (B) At 6 weeks of age. (C) At 6 months of age.

We compared retinal morphology and thickness for ONL, INL and IPL+GCL+NFL obtained by histology and SD-OCT analysis in *Lrit3^+/+^*, *Lrit3^nob6/+^* and *Lrit3^nob6/nob6^* at six weeks and six months of age (Figure 6 and 7A). Examination of histological retinal cross sections showed normal nuclear and synaptic layers among the three genotypes (Figure 6). SD-OCT images exhibited no changes in layer thickness according to the quadrant (dorsal, ventral, temporal or nasal) in each animal. No statistical differences were observed between *Lrit3^+/+^* and *Lrit3^nob6/nob6^* mice in ONL thickness, suggesting normal photoreceptors in the retina of mutant mice (Figure 7B–C). ONL thickness in *Lrit3^nob6/+^* mice was slightly reduced compared to *Lrit3^+/+^* and *Lrit3^nob6/nob6^* mice at 6 weeks (p<0.001) (Figure 7B). However, thickness of INL and IPL+GCL+NFL was statistically decreased in mutant mice compared to *Lrit3^+/+^* and heterozygous (from p<0.001 to p = 0.024) (Figure 7B–C). Thickness of ONL and INL but not IPL+GCL+NFL was also slightly decreased at six months compared to six weeks but these changes were similar in the three genotypes (from p<0.001 to p = 0.025) (Figure 7B–C).

## Discussion

In this work, we have genetically characterized a commercially available *Lrit3* mouse lacking *Lrit3* and determined the impact of this knock-out model on visual function. Our results show that mice lacking *Lrit3* display similar abnormalities as patients with cCSNB due to *LRIT3* mutations and other mice with mutations in genes implicated in the retinal ON-pathway, most strikingly lacking the scotopic ERG b-wave. Here we describe in detail this novel *nob* mouse, which we subsequently called *nob6*.

*nob6* denotes a mouse mutant carrying the *Lrit3* knock-out (ko) allele homozygously. In this ko model, exons 3 and 4 were deleted and replaced by a *Bgeo/Puro* cassette, with only 21 bp of exon 3 still remaining, which was confirmed at genomic and RNA levels. This construction contains a premature stop codon in the first 8 bp of the cassette (c.611_2046delinsGGCCATAG), which is predicted to lead to a truncated 206 amino acid LRIT3 protein (p.Phe204Trpfs*3). A functional antibody against the mouse LRIT3 protein needs to be developed to validate this prediction. If the truncated LRIT3 protein was indeed formed, it would lack several domains, including the transmembrane domain and the PDZ-binding motif that we previously hypothesized to be crucial for LRIT3 function. Our theory would be that LRIT3, with its PDZ-binding motif, might be the missing transmembrane protein, which brings the TRPM1 channel to the cell surface by interacting with scaffolding proteins [21], [23], [43]. Subsequently, the channel opens during light stimulation, allowing the depolarization of ON-bipolar cells, which results in the signal transmission and the ERG b-wave [15], [25], [32]. Without its transmembrane domain and its PDZ-binding motif, LRIT3 could not fulfill its function. TRPM1 would not localize at the dendritic tips of ON-bipolar cells and thus would be inactive. ON-bipolar cells would not depolarize upon light stimulus resulting in the absence of a b-wave. Visual signal transmission would be compromised, leading to night blindness. Interestingly, the *nob6* allele resembles the deletion (c.1538_1539del leading to p.Ser513Cysfs*59) and nonsense mutations (c.1318C>T leading to p.Arg440* and c.1151C>G leading to p.Ser384*) in *LRIT3* identified in cCSNB patients [21]. These three human mutations, located in exon 4, are predicted to lead to truncated proteins lacking the transmembrane domain and the PDZ-binding motif. Thus, the *nob6* mouse represents in fact a reliable model to study the pathological mechanism(s) associated with autosomal recessive complete CSNB due to *LRIT3* mutations.

Functional characterization of *nob6* mouse revealed a stationary *nob* phenotype as found in patients with cCSNB due to *LRIT3* mutations [21], in patients with cCSNB due to other gene defects of the same cascade [5]–[13] and in other mouse models of cCSNB [12], [15], [18], [25], [26], [32], [33], [35], [36], [54]. In scotopic conditions, *nob6* mice defined a selective absence of the ERG b-wave, with a preserved a-wave component. The preserved a-wave indicates that photoreceptors respond normally to light, and the lack of the b-wave localizes the defect to synaptic transmission from the photoreceptors to the ON bipolar cells or to signaling within them. Cone-mediated pathways are also affected since implicit times of both a- and b-waves were delayed and amplitude of b-wave markedly reduced in photopic conditions. Therefore, visual dysfunction in *nob6* mice affects both rod- and cone- ON-bipolar systems. Optomotor tests revealed that, under scotopic conditions, the number of head movements was strongly decreased in *nob6* mice even if the visual acuity is not really measurable [45]. However, we can suppose that, by increasing more the spatial frequency, the optomotor response in mutant mice would reach the zero before wild-type and heterozygous mice and therefore the visual acuity of *nob6* mice would be decreased in scotopic conditions. Moreover, there were no indications for photoreceptor degeneration in this mouse model. Retina morphology investigated by fundus autofluorescence and histology was unsuspicious for all genotypes at the different ages. Similar findings have been reported for other mouse models of cCSNB [12], [18], [26], [33], [35], [36]. However, all steps providing the retinal sections such as fixation, dehydration or cutting are potential sources for significant and variable alteration in dimensions, especially for subtle changes. Thus, an *in vivo* analysis of the retinal structure as SD-OCT may be helpful to detect fine morphological changes without being biased by handling procedures [55]. Moreover, in patients, histology is not possible because it is difficult to have access to human tissues and SD-OCT is used in practice. Indeed, interestingly Godara and co-workers showed that, despite a normal retinal structure using light and electronic microscopy in *nob* mouse mutants carrying mutations in genes coding for postsynaptic proteins, three patients with cCSNB and *GRM6* mutations exhibited a reduced retinal thickness in the extrafoveal region upon SD-OCT examination [56]. This thinning was the result of inner retinal defects, as opposed to photoreceptor loss, and involves GCL. Similarly, as we observed in the *nob6* mice, a normal ONL thickness was measured whereas the inner retinal layer thickness, comprising GCL plus IPL, was reduced. These results support the emerging view that although cCSNB may be considered primarily as a stationary disorder, small structural changes in the retina of patients may be detectable [56]. Thus, this study is the first one using SD-OCT technology to characterize a mouse model with cCSNB. Mutant mice showed no photoreceptor degeneration as measured by the ONL thickness. Interestingly, as Godara and co-workers, we found a small but significant thinning of the inner retina (INL and IPL+GCL+NFL) but it remains unknown if this thinning is functionally relevant. Therefore, it would be interesting to study other patients with cCSNB and the respective mouse models by applying SD-OCT to investigate if this inner retinal thinning is a common finding. We also observed a thinning of ONL and INL between the two time-points but we assumed that this is an ageing consequence because it was observed in the three genotypes and in the same proportions. A slight reduction in ONL thickness was surprisingly detected in heterozygous mice but that phenomenon remains unexplained. In general our data show that the wild-type and heterozygous mice are very similarly, which is what we expected for an autosomal recessive mode of inheritance.

To conclude, this study describes a novel mouse model for human cCSNB associated with *LRIT3* mutations. It creates the basis to clarify the role of LRIT3 in the ON-bipolar cell signaling cascade, presents a tool to confirm putative interactions with various components of this cascade and to elucidate the pathogenic mechanism(s) of cCSNB.

## Supporting Information

Table S1 **Primers used for amplification and sequencing of the flanking intronic and exonic sequences of** ***Lrit3*** **(sequence of reference was purchased by the company Taconic)** Sequences 5′-3′, size of PCR products and annealing temperatures are indicated.
(DOCX)

Table S2 **Primers used for qPCR Taqman on** ***Crb1*** **(NM_133239.2)** c.3481delC p.Arg1161Glyfs*48 is present in *rd8* mouse. Sequences 5′-3′ are indicated.
(DOCX)

Table S3 **Primers used for amplification and sequencing of** ***Pde6β*** **(NM_008806.2)** c.C1041>A p.Tyr347* and Xmv-28 insertion in intron 1 in are present in *rd1* mouse. Sequences 5′-3′, size of PCR products and annealing temperatures are indicated.
(DOCX)

Table S4 **Primers used for amplification and sequencing of** ***Gnat2*** **(NM_008141.2)** c.517G>A p.Asp173Asn is present in *cpfl3* mouse. Sequences 5′-3′, size of PCR products and annealing temperatures are indicated.
(DOCX)

Table S5 **Primers used for amplification and sequencing of** ***Tyr*** **(NM_011661.4)** We tested the c.230G>T p.Arg77Leu mutation. Sequences 5′-3′, size of PCR products and annealing temperatures are indicated.
(DOCX)

Table S6 **Primers used for amplification and sequencing of the flanking intronic and exonic sequences of** ***Grm6*** **(NM_173372.2)** Sequences 5′-3′, size of PCR products and annealing temperatures are indicated.
(DOCX)

Table S7 **Primers used for amplification and sequencing of the flanking intronic and exonic sequences of** ***Gpr179*** **(NM_001081220.1)** Sequences 5′-3′, size of PCR products and annealing temperatures are indicated.
(DOCX)

Table S8 **Primers used for amplification and sequencing of the flanking intronic and exonic sequences of** ***Nyx*** **(AY114303.1)** Sequences 5′-3′, size of PCR products and annealing temperatures are indicated.
(DOCX)

Table S9 **Primers used for amplification and sequencing of the flanking intronic and exonic sequences of** ***Trpm1*** **(NM_001039104.2)** Sequences 5′-3′, size of PCR products and annealing temperatures are indicated.
(DOCX)

Table S10 **Benign** ***Lrit3*** **variants identified in founder mice of** ***Lrit3*** **line (sequence of reference was purchased by the company Taconic)** het: variant found heterozygously; hom: variant found homozygously.
(DOCX)

Table S11 **Benign** ***Trpm1*** **(NM_001039104.2) variants identified in founder mice of** ***Lrit3*** **line** het: variant found heterozygously; hom: variant found homozygously.
(DOCX)
