# Supplementary material for: Lrit3 Deficient Mouse (nob6): A Novel Model of Complete Congenital Stationary Night Blindness (cCSNB)
Source: PLoS One. 2014 Mar 5;9(3):e90342. doi: 10.1371/journal.pone.0090342 (PMC3943948; doi:10.1371/journal.pone.0090342)
Supplement: Table S1 — Primers used for amplification and sequencing of the flanking intronic and exonic sequences of Lrit3 (sequence of reference was purchased by the company Taconic) Sequences 5′-3′, size of PCR products and annealing temperatures are indicated. (DOCX) [file pone.0090342.s001.docx]

| **Primer name** | **Sequence** | **Size of PCR product** | **Annealing temperature** |
| --- | --- | --- | --- |
| mLrit3_1F | CTAAGCTGCTGATGCTAACTG | 365 bp | 60 °C |
| mLrit3_1R | CTGAACCAAGTCTCACGAGG |  |  |
| mLrit3_2aF | CATGCATGTTTCATGTGAGGC | 451 bp | 60 °C |
| mLrit3_2aR | GTTGCTCGACAAGTCCAAGC |  |  |
| mLrit3_2bF | GAGGACGCTGGACTTACACA | 341 bp | 60 °C |
| mLrit3_2bR | GCAGGTGTAAGATCCCAGGT |  |  |
| mLrit3_3F | CTGTCACAAGACAAGCTATGC | 510 bp | 60 °C |
| mLrit3_3R | CCATGTCCTTGCATCCAATGA |  |  |
| mLrit3_3F | CTGTCACAAGACAAGCTATGC | 377 bp | 60 °C |
| mLrit3_CasR | CGACATTCAACAGACCTTGCA |  |  |
| mLrit3_4aF | CACCTTGTAGACAAGTTCCTG | 560 bp | 60 °C |
| mLrit3_4aR | GGTTGCAGAAGCAGCTGAGA |  |  |
| mLrit3_4bF | GGAGGATCAACACCTCCATC | 546 bp | 60 °C |
| mLrit3_4bR | GACACATGCCACATACTGACT |  |  |
| mLrit3_4cF | GAGTCCTCTGTGACTGTGCT | 602 bp | 60 °C |
| mLrit3_4cR | GAGCACATGGAAGTCTCTCC |  |  |
