# Supplementary material for: Lrit3 Deficient Mouse (nob6): A Novel Model of Complete Congenital Stationary Night Blindness (cCSNB)
Source: PLoS One. 2014 Mar 5;9(3):e90342. doi: 10.1371/journal.pone.0090342 (PMC3943948; doi:10.1371/journal.pone.0090342)
Supplement: Table S2 — Primers used for qPCR Taqman on Crb1 (NM_133239.2) c.3481delC p.Arg1161Glyfs*48 is present in rd8 mouse. Sequences 5′-3′ are indicated. (DOCX) [file pone.0090342.s002.docx]

| **Primer name** | **Sequence** |
| --- | --- |
| Crb1-S_taq | CCCTGTTTGCATGGAGGAAA |
| Crb1-AS_taq | CCTGACCATCCCGAGAGACA |
