# Supplementary material for: Lrit3 Deficient Mouse (nob6): A Novel Model of Complete Congenital Stationary Night Blindness (cCSNB)
Source: PLoS One. 2014 Mar 5;9(3):e90342. doi: 10.1371/journal.pone.0090342 (PMC3943948; doi:10.1371/journal.pone.0090342)
Supplement: Table S3 — Primers used for amplification and sequencing of Pde6β (NM_008806.2) c.C1041>A p.Tyr347* and Xmv-28 insertion in intron 1 in are present in rd1 mouse. Sequences 5′-3′, size of PCR products and annealing temperatures are indicated. (DOCX) [file pone.0090342.s003.docx]

| **Primer name** | **Sequence** | **Size of PCR product** | **Annealing temperature** |
| --- | --- | --- | --- |
| Pde6b_7-8F | CATCAGCTTCCTAGCCTCAT | 562 bp | 58 °C |
| Pde6b_7-8R | CTGCTGACCTATCTCTCTAG |  |  |
| Pde6b_G2shortF | TGCTCTGTGGTGTTGCTCTG | 311 bp | 60 °C |
| Pde6b_G1shortR | CTGCATGTGAACCCAGTATTC |  |  |
