# Supplementary material for: Lrit3 Deficient Mouse (nob6): A Novel Model of Complete Congenital Stationary Night Blindness (cCSNB)
Source: PLoS One. 2014 Mar 5;9(3):e90342. doi: 10.1371/journal.pone.0090342 (PMC3943948; doi:10.1371/journal.pone.0090342)
Supplement: Table S4 — Primers used for amplification and sequencing of Gnat2 (NM_008141.2) c.517G>A p.Asp173Asn is present in cpfl3 mouse. Sequences 5′-3′, size of PCR products and annealing temperatures are indicated. (DOCX) [file pone.0090342.s004.docx]

| **Primer name** | **Sequence** | **Size of PCR product** | **Annealing temperature** |
| --- | --- | --- | --- |
| Gnat2_6F | CTAACTCTGTGTTAGCTGTAGA | 631 bp | 60 °C |
| Gnat2_7R | CGAGGTTTGGAGGAGGCTC |  |  |
