# Supplementary material for: Lrit3 Deficient Mouse (nob6): A Novel Model of Complete Congenital Stationary Night Blindness (cCSNB)
Source: PLoS One. 2014 Mar 5;9(3):e90342. doi: 10.1371/journal.pone.0090342 (PMC3943948; doi:10.1371/journal.pone.0090342)
Supplement: Table S5 — Primers used for amplification and sequencing of Tyr (NM_011661.4) We tested the c.230G>T p.Arg77Leu mutation. Sequences 5′-3′, size of PCR products and annealing temperatures are indicated. (DOCX) [file pone.0090342.s005.docx]

| **Primer name** | **Sequence** | **Size of PCR product** | **Annealing temperature** |
| --- | --- | --- | --- |
| Tyr_F | GTTTCCAGATCTCTGATGGC | 320 bp | 58 °C |
| Tyr_R | CAAGACTCGCTTCTCTGTAC |  |  |
