# Supplementary material for: Lrit3 Deficient Mouse (nob6): A Novel Model of Complete Congenital Stationary Night Blindness (cCSNB)
Source: PLoS One. 2014 Mar 5;9(3):e90342. doi: 10.1371/journal.pone.0090342 (PMC3943948; doi:10.1371/journal.pone.0090342)
Supplement: Table S6 — Primers used for amplification and sequencing of the flanking intronic and exonic sequences of Grm6 (NM_173372.2) Sequences 5′-3′, size of PCR products and annealing temperatures are indicated. (DOCX) [file pone.0090342.s006.docx]

| **Primer name** | **Sequence** | **Size of PCR product** | **Annealing temperature** |
| --- | --- | --- | --- |
| Grm6_1F | gcatattgaggatgagtggc | 442 bp | 60 °C |
| Grm6_1R | gcaggaaaagttggtgactc |  |  |
| Grm6_2F | gagtcaccaacttttcctgc | 715 bp | 60 °C |
| Grm6_2R | ctggatgattgggagagattg |  |  |
| Grm6_int2F | ctgagctatccctgaaagca | 574 bp | 60 °C |
| Grm6_int2R | ctgcattagatcagatagcag |  |  |
| Grm6_3F | catctgtgtcacaagtccct | 525 bp | 60 °C |
| Grm6_3R | cagcgtttggaatgcaagac |  |  |
| Grm6_4F | gtcttgcattccaaacgctg | 644 bp | 60 °C |
| Grm6_4R | gatgtgaggagttgctcatg |  |  |
| Grm6_5F | gtagaggtctcctgaagttg | 618 bp | 60 °C |
| Grm6_5R | cagagttactcagcatgtgg |  |  |
| Grm6_6-7F | ctagagatcaccgatatggc | 758 bp | 60 °C |
| Grm6_6-7R | cacttggctcagatatccag |  |  |
| Grm6_8-9aF | ctgcactcattgctaacgag | 771 bp | 60 °C |
| Grm6_8-9aR | GTGAGCAGCACATAGCTAAG |  |  |
| Grm6_8-9bF | CATTGCTACCTTCATGCGAC | 513 bp | 60 °C |
| Grm6_8-9bR | ctgagtgctggtataattgtg |  |  |
| Grm6_10F | cagggaatctaacctgtcag | 597 bp | 60 °C |
| Grm6_10R | ctagaagaacagcgagtgag |  |  |
| Grm6_11F | gtaactcacctccagtctata | 478 bp | 60 °C |
| Grm6_11R | GATGGTCTCTGAGGATCTTTC |  |  |
