# Supplementary material for: Lrit3 Deficient Mouse (nob6): A Novel Model of Complete Congenital Stationary Night Blindness (cCSNB)
Source: PLoS One. 2014 Mar 5;9(3):e90342. doi: 10.1371/journal.pone.0090342 (PMC3943948; doi:10.1371/journal.pone.0090342)
Supplement: Table S7 — Primers used for amplification and sequencing of the flanking intronic and exonic sequences of Gpr179 (NM_001081220.1) Sequences 5′-3′, size of PCR products and annealing temperatures are indicated. (DOCX) [file pone.0090342.s007.docx]

| **Primer name** | **Sequence** | **Size of PCR product** | **Annealing temperature** |
| --- | --- | --- | --- |
| Gpr179_1aF | caggctacatggtactagtg | 777 bp | 60 °C |
| Gpr179_1aR | CCTTAGGTCATTGGTCAGTAC |  |  |
| Gpr179_1bF | GTATCAGGCGCTTGTTCGCA | 617 bp | 60 °C |
| Gpr179_1bR | tgtcttgctcacacgcagca |  |  |
| Gpr179_int1aF | gcactatgcagagaccatttg | 585 bp | 60 °C |
| Gpr179_int1aR | ctggagagtccttgtagagc |  |  |
| Gpr179_int1bF | gacaaggacaggtcttggttc | 728 bp | 60 °C |
| Gpr179_int1bR | ctgtctctgtggtgacagtc |  |  |
| Gpr179_2F | gcagagtaaagaggcggtgt | 721 bp | 60 °C |
| Gpr179_2R | ctggagtgctgagtaccaga |  |  |
| Gpr179_3F | cagagatgagaaagtttccag | 332 bp | 60 °C |
| Gpr179_3R | catagatgttccatctcgact |  |  |
| Gpr179_4F | gctagaccttcacgtgtctc | 532 bp | 60 °C |
| Gpr179_4R | ctcttgcagagtgcctctgt |  |  |
| Gpr179_5F | cagagtgagcatcctgacag | 430 bp | 60 °C |
| Gpr179_5R | ctgtgacatctgggatgtac |  |  |
| Gpr179_6-7F | gacatgtgaagcctctactg | 690 bp | 60 °C |
| Gpr179_6-7R | CAGTGGTCATGGTGACAGAG |  |  |
| Gpr179_7-8F | CAGCTCCTGCTGCTGGTATT | 781 bp | 60 °C |
| Gpr179_7-8R | gctctgaagcaaaggctaag |  |  |
| Gpr179_9F | cagagagtcagtgaggtgag | 361 bp | 60 °C |
| Gpr179_9R | ctagctctatgttgcttgtga |  |  |
| Gpr179_10F | gactctgaaggtgtggaatg | 764 bp | 60 °C |
| Gpr179_10R | ctgtgttcctctgctcagtc |  |  |
| Gpr179_11aF | gatacaacttggtggagaagt | 623 bp | 60 °C |
| Gpr179_11aR | ACACTGAGTGACTTCTGCAG |  |  |
| Gpr179_11bF | CTCAGCTCCAGCCTTCAAGA | 732 bp | 60 °C |
| Gpr179_11bR | CAGATGAAAGTGAGAAGGGTG |  |  |
| Gpr179_11cF | CATTGGCTCCTATTCTGTTGC | 780 bp | 60 °C |
| Gpr179_11cR | GATTCCTTCGCTTGTTCAGC |  |  |
| Gpr179_11dF | CAGCCGAGAGCAAGAAGATA | 654 bp | 60 °C |
| Gpr179_11dR | GTACCCTCTGAGTAGATGTC |  |  |
| Gpr179_11eF | CATCAGGAGTCTGACAGAAC | 671 bp | 60 °C |
| Gpr179_11eR | CAAGGACACACTTGCAGTAC |  |  |
| Gpr179_11fF | CTCAAGGACAGGAGTCAGTA | 686 bp | 60 °C |
| Gpr179_11fR | CTCAGTGTTCGGAGTGTCTG |  |  |
| Gpr179_11gF | GCGTCTACAGATTCTGGATC | 630 bp | 60 °C |
| Gpr179_11gR | CTGAAGTTCCGAGCTTGCTC |  |  |
| Gpr179_11hF | GAGAGCTGAGACAAGACCTG | 684 bp | 60 °C |
| Gpr179_11hR | CTGTCTCTGCTGCCTTCAAG |  |  |
| Gpr179_11iF | CTATCAAGGAAGCTGAGATCT | 567 bp | 60 °C |
| Gpr179_11iR | GAACTGACTTGGCTCCAGTC |  |  |
| Gpr179_11jF | CAGTCTGTCTCTCGGTACAC | 406 bp | 60 °C |
| Gpr179_11jR | GTGCTGTCATTCACAGTCTC |  |  |
