# Supplementary material for: Lrit3 Deficient Mouse (nob6): A Novel Model of Complete Congenital Stationary Night Blindness (cCSNB)
Source: PLoS One. 2014 Mar 5;9(3):e90342. doi: 10.1371/journal.pone.0090342 (PMC3943948; doi:10.1371/journal.pone.0090342)
Supplement: Table S8 — Primers used for amplification and sequencing of the flanking intronic and exonic sequences of Nyx (AY114303.1) Sequences 5′-3′, size of PCR products and annealing temperatures are indicated. (DOCX) [file pone.0090342.s008.docx]

| **Primer name** | **Sequence** | **Size of PCR product** | **Annealing temperature** |
| --- | --- | --- | --- |
| Nyx_1F | gactcctgctgattcagtag | 579 bp | 60°C |
| Nyx_1R | catagacgttgaaggagcaat |  |  |
| Nyx_2aF | caacttaatggatcacctgtc | 785 bp | 60°C |
| Nyx_2aR | CTGAACTGGTCTAACCTCTG |  |  |
| Nyx_2bF | CATGTTCCCACCTTGATGCT | 780 bp | 60°C |
| Nyx_2bR | GCAAACTGCTGATtTCATCTC |  |  |
| Nyx_2cF | GACACAGGACTTAATGGCC | 745 bp | 60°C |
| Nyx_2cF | aggtatcactcaggacccag |  |  |
| Nyx_3F | cttggagatgggccattaag | 563 bp | 60°C |
| Nyx_3F | gtcacagttaccacttacttg |  |  |
| Nyx_4aF | gcatacagtgttctctgtatc | 726 bp | 60°C |
| Nyx_4aR | GCGGAAGAGATTGTCGAAGG |  |  |
| Nyx_4bF | CACAACGGTGAGCTGCGCT | 670 bp | 62°C |
| Nyx_4bR | AGTCACAGCGCCAAGGATTG |  |  |
| Nyx_4cF | CTGCATCTCAATGGCAACCG | 604 bp | 60°C |
| Nyx_4cR | CAGGCAGTTACTCATGGCAG |  |  |
