# Supplementary material for: Lrit3 Deficient Mouse (nob6): A Novel Model of Complete Congenital Stationary Night Blindness (cCSNB)
Source: PLoS One. 2014 Mar 5;9(3):e90342. doi: 10.1371/journal.pone.0090342 (PMC3943948; doi:10.1371/journal.pone.0090342)
Supplement: Table S9 — Primers used for amplification and sequencing of the flanking intronic and exonic sequences of Trpm1 (NM_001039104.2) Sequences 5′-3′, size of PCR products and annealing temperatures are indicated. (DOCX) [file pone.0090342.s009.docx]

| **Primer name** | **Sequence** | **Size of PCR product** | **Annealing temperature** |
| --- | --- | --- | --- |
| Trpm1_1F | CCATTTGCTAAATCTGCCTCC | 602 bp | 60 °C |
| Trpm1_1R | CCACGGCTACCAGTACAAC |  |  |
| Trpm1_2F | GTGTTCATGTGTCATGGTTCA | 549 bp | 58 °C |
| Trpm1_2R | GATCTTGCTCACATCTGGCT |  |  |
| Trpm1_3F | CATGCAAAGTGTTGCAAGCAG | 687 bp | 60 °C |
| Trpm1_3R | CATGTGTGCAAGGGTAGGCT |  |  |
| Trpm1_4F | GCCAAAAACCGTGTACCTTG | 658 bp | 58 °C |
| Trpm1_4R | CAGTTGGGATCAGGCTTCC |  |  |
| Trpm1_5F | GAGACCCAGTGACTTCCAG | 568 bp | 58 °C |
| Trpm1_5R | GATGGCAGCAATGTCAGCAT |  |  |
| Trpm1_6F | GGCTGCCACTGTCTAAAGC | 757 bp | 58 °C |
| Trpm1_6R | GGTGGCTAGGGCAAAGAGT |  |  |
| Trpm1_7F | CAGTCACCCTTCGCTTGAC | 491 bp | 58 °C |
| Trpm1_7R | CCTGGGCATTCTCATGCTG |  |  |
| Trpm1_8F | CAGCATGAGAATGCCCAGG | 555 bp | 58 °C |
| Trpm1_8R | GCACTGGCAGCTAGTTTGG |  |  |
| Trpm1_9F | GCAGGATGATAGATTCCAATC | 493 bp | 58 °C |
| Trpm1_9R | CAGAGTCTGCTCATCCCAG |  |  |
| Trpm1_10F | CAGTTGGCTTGGGATGAGG | 600 bp | 58 °C |
| Trpm1_10R | CTACATCATGAGCAAGGACC |  |  |
| Trpm1_11F | GCACATAGAGTTCAGGGTGT | 472 bp | 58 °C |
| Trpm1_11R | CTCCTCCTTACGAAACAGCT |  |  |
| Trpm1_12F | CCATGGGCTCAATTTCCAGC | 665 bp | 60 °C |
| Trpm1_12R | GTCGTGATGTTCAGTGGGCT |  |  |
| Trpm1_13F | CTACAGGCTCCACATTTCAG | 365 bp | 58 °C |
| Trpm1_13R | CCAACTTCAGTTCTCACGCT |  |  |
| Trpm1_14F | GGCTGATCTAGGGTATGTGA | 531 bp | 58 °C |
| Trpm1_14R | GAGTTGCATTCTGCACAGGT |  |  |
| Trpm1_15F | GCGTTGACTCACCTGGATG | 474 bp | 58 °C |
| Trpm1_15R | GGTGACAACCTCTGACCTG |  |  |
| Trpm1_16F | CTTCTGACCCGGAAGTCTC | 770 bp | 58 °C |
| Trpm1_16R | GACCACCAAGGGCCTAATC |  |  |
| Trpm1_17F | CTTGTGGCTCCTCAAGTTAC | 558 bp | 58 °C |
| Trpm1_17R | GCACGCAGATGGTGTATGTT |  |  |
| Trpm1_18F | CAGGATGTGAGCTGCTTTGC | 454 bp | 60 °C |
| Trpm1_18R | GGTGGACCTTTCCAGCACAA |  |  |
| Trpm1_19F | TCCACTTTGTGCTGGAAAGGT | 826 bp | 60 °C |
| Trpm1_19R | GCTCTCAGCTGGTCCTAGTT |  |  |
| Trpm1_20F | CAGAGCCTGTACTGCACCAA | 556 bp | 60 °C |
| Trpm1_20R | GCAGCATTTGGTGTAGCTCG |  |  |
| Trpm1_21F | CTGACTGTCCTGGCACTCTT | 519 bp | 60 °C |
| Trpm1_21R | CCATGCCTTCCTGTTGGAGT |  |  |
| Trpm1_22F | CAGTGGATGAGAAGGGACGA | 646 bp | 60 °C |
| Trpm1_22R | GATAGTCCTGGTCTGGCGT |  |  |
| Trpm1_23F | CATTGCTTCCCAGCATCAGC | 462 bp | 60 °C |
| Trpm1_23R | CTGCCACCCTCTCAGATCAA |  |  |
| Trpm1_24F | GTCTGGCCTCCACATACAGA | 595 bp | 60 °C |
| Trpm1_24R | TCAGGAGACTTCTGCCTATGA |  |  |
| Trpm1_25F | CACCAGCTCATCTACAGCGT | 602 bp | 60 °C |
| Trpm1_25R | GCTGAGGTTGCTATGGAAGTG |  |  |
| Trpm1_26F | CTCAATGGCACAGCTAAACC | 498 bp | 58 °C |
| Trpm1_26R | CTGGCTTGGCAAAAGCAACA |  |  |
| Trpm1_27aF | CATGAACAAACAGGCCCAGC | 774 bp | 60 °C |
| Trpm1_27aR | GTAGCGCACGAGTTTGCTCT |  |  |
| Trpm1_27aF | GGAAGCTGCACCAAGTCTGA | 698 bp | 60 °C |
| Trpm1_27aR | GTCAAGATGGCAGCCATCGA |  |  |
| Trpm1_10Fseq | GAAAGGAGGAAGTGATAGGTG |  | 60 °C |
