# Supplementary material for: Lrit3 Deficient Mouse (nob6): A Novel Model of Complete Congenital Stationary Night Blindness (cCSNB)
Source: PLoS One. 2014 Mar 5;9(3):e90342. doi: 10.1371/journal.pone.0090342 (PMC3943948; doi:10.1371/journal.pone.0090342)
Supplement: Table S10 — Benign Lrit3 variants identified in founder mice of Lrit3 line (sequence of reference was purchased by the company Taconic) het: variant found heterozygously; hom: variant found homozygously. (DOCX) [file pone.0090342.s010.docx]

| **SNP** | **Exon** | **Nucleotide Exchange** | **Allele State** | **Protein Effect** | **Alleles Frequency (UCSC) and comments** |
| --- | --- | --- | --- | --- | --- |
| rs225142634 | Intron1 | c.116+6_116+9insTATG | het 3, hom 3 |  | found in 129 substrains: 129S5 (ss433756752), 129S1 (ss435114887), 129P2 (ss434329892) (Ensembl) |
| rs30623771 | Intron1 | c.116+9T>C | het 3, hom 3 |  | more G than A (UCSC), A in C57BL/6NJ; G in 129P2/OlaHsd, 129S1/SvImJ, 129S5SvEvBrd (Ensembl) |
| rs30621348 | 2 | c.267T>G | het 3, hom 3 | p.(=) | A: 75.000% (6 / 8); C: 25.000% (2 / 8), A in C57BL/6NJ, C57BL/6J; G in 129P2/OlaHsd, 129S1/SvImJ, 129S5SvEvBrd, 129X1/SvJ (Ensembl) |
| rs30670584 | 2 | c.299G>C | het 3, hom 3 | p.Ser100Thr | G: 25.000% (2 / 8); C: 75.000% (6 / 8), well conserved and no Threonine found (USCS), G in 129P2/OlaHsd, 129S1/SvImJ, 129S5SvEvBrd, 129X1/SvJ; C in C57BL/6NJ, C57BL/6J (Ensembl), probably damaging (PolyPhen-2), tolerated (SIFT) |
| rs30628816 | 2 | c.549G>A | het 3, hom 3 | p.(=) | T: 25.000% (2 / 8); C: 75.000% (6 / 8), C in C57BL/6NJ, C57BL/6J; T in 129P2/OlaHsd, 129S1/SvImJ, 129S5SvEvBrd, 129X1/SvJ (Ensembl) |
| rs30622656 | 3 | c.664G>T | hom 3 | p.Ala222Ser | quite conserved and Serine found in dog, turkey, chicken, zebra finch, budger igar and lizard (UCSC), C in C57BL/6NJ; A in 129P2/OlaHsd, 129S1/SvImJ, 129S5SvEvBrd (Ensembl), benign (PolyPhen-2), tolerated (SIFT) |
| rs30356146 | 3 | c.777A>G | hom 3 | p.(=) | T: 66.667% (4 / 6); C: 33.333% (2 / 6), T in C57BL/6NJ, C57BL/6J; C in 129P2/OlaHsd, 129S1/SvImJ, 129S5SvEvBrd, 129X1/SvJ (Ensembl) |
| rs30621805 | 4 | c.922A>G | hom 3 | p.Ile308Val | more C than T (UCSC), T in C57BL/6NJ; C in 129P2/OlaHsd, 129S1/SvImJ, 129S5SvEvBrd (Ensembl), benign (PolyPhen-2), tolerated (SIFT) |
| rs264628066 | 4 | c.949A>G | hom 3 | p.Ser317Gly | more C than T (UCSC), T in C57BL/6NJ; C in 129P2/OlaHsd, 129S1/SvImJ, 129S5SvEvBrd (Ensembl), benign (PolyPhen-2), tolerated (SIFT) |
| rs30620873 | 4 | c.1008T>G | hom 3 | p.Ile336Val | more C than A (UCSC), A in C57BL/6NJ; C in 129P2/OlaHsd, 129S1/SvImJ, 129S5SvEvBrd (Ensembl), benign (PolyPhen-2), tolerated (SIFT) |
| rs231314865 | 4 | c.1025C>G | hom 3 | p.Thr342Ser | well conserved but C found in opossum, tasmanian devil, wallaby and lizard (UCSC), C in 129P2/OlaHsd, 129S1/SvImJ, 129S5SvEvBrd; G in C57BL/6NJ (Ensembl), benign (PolyPhen-2), tolerated (SIFT) |
| rs30620871 | 4 | c.1485T>C | hom 3 | p.(=) | A in C57BL/6NJ; G in 129P2/OlaHsd, 129S1/SvImJ, 129S5SvEvBrd (Ensembl) |
| rs30620869 | 4 | c.1511T>C | hom 3 | p.Met504Thr | quite conserved and many Threonine found (UCSC), A in C57BL/6NJ; G in 129P2/OlaHsd, 129S1/SvImJ, 129S5SvEvBrd (Ensembl), benign (PolyPhen-2),  tolerated (SIFT) |
| rs224939564 | 4 | c.1531_1533insAGC | hom 3 | p.Ser511_Thr512insSer | found in 129 substrains: 129S1 (ss435114809), 129P2 (ss434329773), 129S5 (ss433756614) (Ensembl) |
