# Supplementary material for: Lrit3 Deficient Mouse (nob6): A Novel Model of Complete Congenital Stationary Night Blindness (cCSNB)
Source: PLoS One. 2014 Mar 5;9(3):e90342. doi: 10.1371/journal.pone.0090342 (PMC3943948; doi:10.1371/journal.pone.0090342)
Supplement: Table S11 — Benign Trpm1 (NM_001039104.2) variants identified in founder mice of Lrit3 line het: variant found heterozygously; hom: variant found homozygously. (DOCX) [file pone.0090342.s011.docx]

| **SNP** | **Exon** | **Nucleotide Exchange** | **Allele State** | **Protein Effect** | **Alleles Frequency (UCSC) and comments** |
| --- | --- | --- | --- | --- | --- |
| rs52569084 | 11 | c.1375A>G | het | p.Thr459Ala | quite conserved and Alanine found in rat and guinea pig (UCSC), A in 129P2/OlaHsd, C57BL/6NJ; G in 129S1/SvImJ, 129S5SvEvBrd, benign (Polyphen-2), tolerated (SIFT) |
| rs37965510 | 27 | c.4309G>A | het 5, hom 1 | p.Asp1437Asn | Minor Allele Frequency is at least 5% in all populations assayed, not conserved but no Aspargine found (UCSC), G in 129P2/OlaHsd, C57BL/6NJ; A in 129S1/SvImJ, 129S5SvEvBrd (Ensembl), benign (Polyphen-2), damaging but with low confidence (SIFT) |
| rs32166708 | 27 | c.4441A>G | het | p.Ser1481Gly | G: 50.000% (4 / 8); A: 50.000% (4 / 8), quite conserved and Glycine found in rat, kangaroo rat, guinea pig, microbat, armadillo (UCSC), A in C57BL/6NJ, C57BL/6J; G in 129P2/OlaHsd, 129S1/SvImJ, 129S5SvEvBrd, 129X1/SvJ (Ensembl), benign (Polyphen-2), tolerated (SIFT) |
